# Analytical solution of fuzzy heat problem in two-dimensional case under Caputo-type fractional derivative

**DOI:** 10.1371/journal.pone.0301719

**Published:** 2024-04-19

**Authors:** Muhammad Nadeem, Chen Yilin, Devendra Kumar, Yahya Alsayyad

**Affiliations:** 1 School of Mathematics and Statistics, Qujing Normal University, Qujing, China; 2 Department of Mathematics, University of Rajasthan, Jaipur, Rajasthan, India; 3 Department of Physics, Hodeidah University, Al-Hudaydah, Yemen; The British University in Egypt, EGYPT

## Abstract

This work aims to investigate the analytical solution of a two-dimensional fuzzy fractional-ordered heat equation that includes an external diffusion source factor. We develop the Sawi homotopy perturbation transform scheme (SHPTS) by merging the Sawi transform and the homotopy perturbation scheme. The fractional derivatives are examined in Caputo sense. The novelty and innovation of this study originate from the fact that this technique has never been tested for two-dimensional fuzzy fractional ordered heat problems. We presented two distinguished examples to validate our scheme, and the solutions are in fuzzy form. We also exhibit contour and surface plots for the lower and upper bound solutions of two-dimensional fuzzy fractional-ordered heat problems. The results show that this approach works quite well for resolving fuzzy fractional situations.

## 1 Introduction

Over the past thirty years, the fractional calculus (FC) study has attracted a lot of interest. The majority of the scientists have contributed to this topic by incorporating multiple operators with fractional numbers in various works. Modern calculus yielded more realistic results than traditional calculus. The structures of numerous situations in practical life involving two integers were described by FC. In addition, fractional operators provided more degrees of freedom than integer differential operators [1, 2]. Numerous investigators have examined the phenomenon of fractional calculus in several valuable fields of engineering and science. The investigation of geometrical and physical foundations of fractional-order derivatives was first presented by Podlubny [3]. Diethelm and Ford [4] investigated the dynamical results of the fractional order problems under the different operators. Kumar et al. [5] investigated the complicated behavior of a dynamical structure using fractional and fractal-fractional derivative operators and showed that non-classical derivatives are particularly effective in examining the hidden behavior of the systems. Many researchers employed the fundamental principles and properties of operators given within the context of FC to examine simulations showing viruses, bifurcation, chaos, control theory, image processing, quantum fluid flow, and several other related areas [6–8].

Fuzzy set theory is an effective technique for simulating unpredictable challenges. This has led to the modeling of a wide range of natural phenomena using fuzzy laws. The fuzzy fractional differential equation is a popular framework in a variety of scientific domains, including population modeling, physical science, mechanical work, chemical reactions, and civil engineering. As a result, the study of fuzzy calculus has gained attraction in the field of fractional derivatives. The concept of fuzzy set was associated with geometrical function theory in 2011 with the introduction of fuzzy subordination [9]. Agarwal et al. [10] considered the fuzzy differential equations by incorporating it into the dynamical system with uncertainty for the first time. Van Hoa [11] investigated the existence and uniqueness of solutions to fuzzy fractional functional differential equations with Caputo generalized Hukuhara differentiability. Long et al. [12] present two new results on the existence of generalized Hukuhara-weak solutions fuzzy fractional partial differential equations. One depends on the Banach fixed point theorem with the Lipschitz condition and the other is depending on a nonlinear alternate on the Schauder type fuzzy-valued continuous functions without the Lipschitz condition. Salahshour et al. [13] considered fuzzy Laplace transforms for the solution of fuzzy fractional differential equations under Riemann-Liouville H-differentiability. Iqbal et al. [14] proposed an iterative transform method for the approximate solution of fractional fuzzy acoustic wave model.

Fuzzy integral equations have several applications in various practical problems such as industrial engineering, scientific computing, physical sciences and neural network. It is studied that the existing study problem with fractional order derivatives can be turned to uncertain problems [15, 16]. As a result, several scholars focused on such frameworks in order to examine their solutions analytically or numerically. In [17], authors discussed existence, uniqueness and numerical examination under fractional-order ideas and obtained the analytical results of several linear and nonlinear fuzzy fractional models. Arfan et al [18] developed an algorithm based on the HPS to analyze the analytical results for two dimensional fuzzy fractional heat problem consisting of external source term. [19] introduced two different schemes to find out the approximate and analytical results of fuzzy fractional problems. Hamoud and Ghadle [20] considered the homotopy analysis strategy and obtained the solution of the first order fuzzy Volterra-Fredholm integro-differential equations. Ali and Hadhoud [21] used Haar wavelet scheme to derive the series solution of nonlinear fuzzy integro-differential problems. In [22], authors provided the differential inclusions idea for the solution of fuzzy problems. Arqub and Al-Smadi [23] discussed the singularity, existence, and other features of fuzzy conformable fractional differential problems.

Consider the two-dimensional fuzzy fractional heat problem such as

$$\begin{array}{c} D_{t}^{\alpha }\tilde{\vartheta }\left(x,y,t\right)=D_{x}^{2}\tilde{\vartheta }\left(x,y,t\right)+D_{y}^{2}\tilde{\vartheta }\left(x,y,t\right)+g\left(x,y,t\right), \end{array}$$ (1)

with initial condition

$$\begin{array}{c} \tilde{\vartheta }\left(x,y,0\right)=\tilde{f}\left(x,y\right), \end{array}$$ (2)

where *α* represents the Caputo fractional derivative and g∈C([0,∞)×[0,∞)×[0,∞),[0,∞)), $\tilde{f}\in \left(\left[0,\infty \right)\times \left[0,\infty \right),\left[0,\infty \right)\right)$. It is pointed out that, 2D heat problem represents the heat transfer through an infinite thin sheet. In Eq (1), the term “$\tilde{\vartheta }$” represents the temperature of a particle at instance spot within a very small sheet. This phenomenon of heat changing can study to various discipline of science and engineering. Therefore, the analysis of two-dimensional fuzzy fractional heat equations has much more application in various domains, such as heat transfer analysis in materials with uncertain properties, modeling of temperature distribution in environmental systems, or analysis of thermal processes in complex systems with imprecise parameters.

The Homotopy perturbation scheme (HPS) was first proposed by He [24], which is the combination of the homotopy scheme and classical perturbation technique. In recent years, many researchers [25–27] studied the multiple forms of linear and nonlinear differential problems. Sene and Fall [28] used homotopy perturbation Laplace transform method to obtain the approximate solution of fractional diffusion equation and the fractional diffusion-reaction equation. The significance feature of HPS is that it provides better performance when it is coupled with other techniques to obtain the approximations of fractional challenges such as Lal and Vir [29] presented the coupled scheme of Laplace transform and HPS for the solution of Fokker-Planck problems. Jani and Singh [30] coupled Abdooh transform with HPS in order to obtain the solution of fractional order atmospheric internal waves model. Elzaki and Biazar [31] proposed a method by combining Elzaki transform and HPS to achieve the analytical results of nonlinear partial differential equations. Maitama and Zhao [32] proposed homotopy perturbation Shehu transform method to solve wave-like fractional models and obtained the closed form results. The efficiency of HPS in solving homogeneous and nonhomogeneous partial differential equations is also shown in [33–35].

In this study, we combined the Sawi transform and the homotopy perturbation scheme to determine an approximation for a two-dimensional fuzzy fractional heat problem. The key advantages of this strategy is that it does not require any assumption in main problem, so it overcomes the impediments of the classical perturbation technique and consumes less time in the truncated series. This method gives a power series results in the sense of rapid convergent series that leads the high accuracy only a few iterations. The SHPTS does not involve rounding errors, assuming linearization, perturbation, or descretization. In addition, the proposed scheme can overcome the fractional order by utilizing He’s polynomials in dealing with difficult terms of the problem. The numerical results demonstrate that this technique remains more robust, convergence, and straightforward compared to other numerical approaches. The proposed scheme demonstrates how effective the applied strategy is in obtaining the solutions for supplied local fractional partial differential equations. Some visualizations are also provided to demonstrate its performance in the presence of graphical limitations. We introduce the concept of the Sawi transform and provide definitions in Section (2). In Sections (3) and (4), we expand the concepts of HPS and SHPTS for fractional problem with lower and upper bound. Section (5) includes an explanation of the convergence theorem. We demonstrate several examples in Section (6) to verify the efficiency and validity of suggested approach. We summarize the conclusion in final Section (7).

## 2 Preliminaries

This section presents the concepts of Sawi transform along its some basic properties that are required during the development of the SHPTS.

**Definition 2.1** The Sawi transform is defined such as [36, 37]

$$\begin{array}{c} S\left[\vartheta \left(℘\right)\right]=ℜ\left(\theta \right)=\frac{1}{\theta^{2}}\int_{0}^{\infty }\vartheta \left(℘\right)e^{-\frac{℘}{\theta }}dt,\;\;\;℘\geq 0,\;\;\;k_{1}\leq \theta \leq k_{2}, \end{array}$$

where *θ* is the transform variable. If *ϑ*(**℘**) is piecewise continuous and of exponential order, the ST of the function *ϑ*(**℘**), **℘** ≥ 0 exist otherwise ST may or may not exist. If *R*(*θ*) is the ST of *ϑ*(**℘**) then *ϑ*(**℘**) is the inverse of *R*(*θ*) such that,

$$\begin{array}{c} S^{-1}\left[ℜ\left(\theta \right)\right]=\vartheta \left(℘\right),\;\;\;\;\;S^{-1}\;\text{is}\;\text{called}\;\text{the}\;\text{inverse}\;\text{Sawi}\;\text{transform}. \end{array}$$

**Definition 2.2** If *S*{*ϑ*_1_(**℘**)} = ℜ_1_(*θ*) and *S*{*ϑ*_2_(**℘**)} = ℜ_2_(*θ*), then [38, 39]

$$\begin{array}{c} S\left\{a_{1}\vartheta_{1}\left(℘\right)+a_{2}\vartheta_{2}\left(℘\right)\right\}=S\left\{a_{1}\vartheta_{1}\left(℘\right)\right\}+a_{2}S\left\{\vartheta_{2}\left(℘\right)\right\}, \end{array}$$

that yields the linear property as

$$\begin{array}{c} S\left\{a_{1}\vartheta_{1}\left(℘\right)+ba_{2}\vartheta_{2}\left(℘\right)\right\}=a{ℜ}_{1}\left(℘\right)+b{ℜ}_{2}\left(℘\right). \end{array}$$

where *a* and *b* are arbitrary constants.

**Definition 2.3** Since *S*{*ϑ*(**℘**)} = ℜ(*θ*), then the following properties can be stated as

**a)** $S\left\{\vartheta^{\prime }\left(℘\right)\right\}=\frac{ℜ(\theta )}{\theta }-\frac{\vartheta (0)}{\theta^{2}},$

**b)** $S\left\{\vartheta^{\prime \prime }\left(℘\right)\right\}=\frac{ℜ(\theta )}{\theta^{2}}-\frac{\vartheta (0)}{\theta^{3}}-\frac{\vartheta^{\prime }\left(0\right)}{\theta^{2}},$

**c)** $S\left\{\vartheta^{m}\left(℘\right)\right\}=\frac{ℜ(\theta )}{\theta^{m}}-\frac{\vartheta (0)}{\theta^{m+1}}-\frac{\vartheta^{\prime }\left(0\right)}{\theta^{m}}-\cdots -\frac{\vartheta^{m-1}\left(0\right)}{\theta^{2}}.$

**Definition 2.4** The Caputo’s derivative of *ϑ*(**℘**) under the fractional-order is expressed as,

$$\begin{array}{cc} D_{℘}^{\alpha }\vartheta \left(℘\right)= & \frac{1}{\Gamma (n-\alpha )}\int_{0}^{℘}{\left(℘-s\right)}^{n-\alpha -1}\vartheta^{n}\left(s\right)\;ds,\;n-1<\alpha \leq n.\;n\in N \end{array}$$

**Definition 2.5** The Sawi transform in fractional derivative is given as

$$\begin{array}{c} S\left[D_{℘}^{\alpha }\vartheta \left(℘\right)\right]=\frac{1}{\theta^{\alpha }}S\left[\vartheta \left(℘\right)\right]-\sum_{k=0}^{n-1}{\left(\frac{1}{\theta }\right)}^{\alpha -\hbar +1}\vartheta^{k}\left(0\right). \end{array}$$

**Definition 2.6** [40] A fuzzy number *u* ∈ *E*^1^ is called to be positive if $\underline{u}\left(1\right)\geq 0$, strict positive if $\underline{u}\left(1\right)>0$, negative if $\bar{u}\left(1\right)\leq 0$ and strict negat ive if $\bar{u}\left(1\right)<0$. The set of positive (negative) fuzzy numbers is denoted by $E_{+}^{1}(E_{-}^{1})$.

**Definition 2.7** The lower and upper bounds of all fuzzy numbers must satisfy the following conditions [41]

- (i) $\underline{k}\left(r\right)$ is a nondecreasing bounded left-continuous function over the interval [0, 1].
- (ii) $\bar{k}\left(r\right)$ is a nonincreasing bounded right-continuous function over the interval [0, 1].
- (iii) $\underline{k}\left(r\right)\leq \bar{k}\left(r\right),\;0\leq r\leq 1$.

if $\underline{k}\left(r\right)=\bar{k}\left(r\right)=r$, where *r* is the crisp factor.

**Theorem 2.1** Suppose there are two positive fuzzy numbers *u* and *v* such that *ϑ* = *uv* where *ϑ*(*r*) = $\left[\underline{\vartheta }\left(r\right),\bar{\vartheta }\left(r\right)\right]$. The following properties are true [42]

$$\begin{array}{c} \underline{\vartheta }\left(r\right)=\underline{u}\left(r\right)\underline{v}\left(1\right)+\underline{u}\left(1\right)\underline{v}\left(r\right)-\underline{u}\left(1\right)\underline{v}\left(1\right), \end{array}$$

and

$$\begin{array}{c} \bar{\vartheta }\left(r\right)=\bar{u}\left(r\right)\bar{v}\left(1\right)+\bar{u}\left(1\right)\bar{v}\left(r\right)-\bar{u}\left(1\right)\bar{v}\left(1\right), \end{array}$$

in which *r* ∈ [0, 1] is a fuzzy positive digit. Let *ϑ* be a fuzzy-valued function, and let *D* be its domain. Let us define $\underline{\vartheta }\left(\ldots ,r\right),\bar{\vartheta }\left(.,.,r\right):D\to R$ for all 0 ≤ *r* ≤ 1. These functions are known as the left and right *r*-level functions of *ϑ*.

**Theorem 2.2** Let $\vartheta :R_{+}\times R_{+}\to E^{1}$ be a continuous fuzzy-valued function. Suppose the functions $\frac{1}{\sigma^{2}}e^{-\frac{℘}{\sigma }}\vartheta \left(ℑ,℘\right),\frac{1}{\sigma^{2}}e^{-\frac{℘}{\sigma }}\frac{\partial^{n}\vartheta \left(ℑ,℘\right)}{\partial {ℑ}^{n}}$ are inappropriate fuzzy Riemann-integrable in terms of ℘ on [0, ∞). Subsequently, the coming fact are

$$\begin{array}{c} S[\frac{\partial^{n}\vartheta \left(ℑ,℘\right)}{\partial {ℑ}^{n}}]=\frac{\partial^{n}}{\partial {ℑ}^{n}}S\left[\vartheta \left(ℑ,℘\right)\right], \end{array}$$

where *S*[*ϑ*(ℑ, **℘**)] shows the Sawi transform of function *ϑ*.

**Proof.** Since *ϑ*(ℑ, **℘**) be (i)-differentiable and from above equation, we can have

$$\begin{array}{l} S[\frac{\partial^{n}\vartheta (ℑ,℘)}{\partial {ℑ}^{n}}]=(FR)\int_{0}^{\infty }\frac{1}{\sigma^{2}}e^{-\frac{℘}{\sigma }}⊙\frac{\partial^{n}\vartheta (ℑ,℘)}{\partial {ℑ}^{n}}d℘\\ =\left(\int_{0}^{\infty }\frac{1}{\sigma^{2}}e^{-\frac{℘}{\sigma }}\frac{\partial^{n}\underline{\vartheta }(ℑ,℘,r)}{\partial {ℑ}^{n}}d℘,\int_{0}^{\infty }\frac{1}{\sigma^{2}}e^{-\frac{℘}{\sigma }}\frac{\partial^{n}\bar{\vartheta }(ℑ,℘,r)}{\partial {ℑ}^{n}}d℘\right)\\ =\frac{\partial^{n}}{\partial {ℑ}^{n}}\left(\int_{0}^{\infty }\frac{1}{\sigma^{2}}e^{-\frac{℘}{\sigma }}\underline{\vartheta }(ℑ,℘,r)d℘,\int_{0}^{\infty }\frac{1}{\sigma^{2}}e^{-\frac{℘}{\sigma }}\bar{\vartheta }(ℑ,℘,r)d℘\right)=\frac{\partial^{n}}{\partial {ℑ}^{n}}S[\vartheta (ℑ,℘)]. \end{array}$$

**Lemma 2.3** Consider $\tilde{\vartheta }$ on [0, *b*] is a subest of region *R* and a continues fuzzy parameter. The fuzzy fractional integral associated with **℘** in the Riemann-Liouville theory is expressed as [18]

$$\begin{array}{c} I^{\zeta }\tilde{\vartheta }\left(℘\right)=\int_{0}^{℘}\frac{{\left(℘-\phi \right)}^{\zeta -1}\tilde{\vartheta }\left(\phi \right)}{\Gamma (\zeta )}d\zeta ,\phi \in \left(0,\infty \right). \end{array}$$

Further, if $\tilde{\vartheta }\in C^{F}\left[0,b\right]\cap L^{F}\left[0,b\right]$, in which *C*^*F*^[0, *b*] is fuzzy continues space functions and *L*^*F*^[0, *b*] is fuzzy Lebesgue integrable space functions, then we have

$$\begin{array}{c} {\left[I^{\zeta }\tilde{\vartheta }\left(℘\right)\right]}_{r}=[I^{\zeta }{\underline{\vartheta }}_{r}\left(℘\right),I^{\zeta }{\bar{\vartheta }}_{r}\left(℘\right)],\;0\leq r\leq 1, \end{array}$$

thus

$$\begin{array}{c} \begin{array}{cc} & I^{\zeta }{\underline{\vartheta }}_{r}\left(℘\right)=\int_{0}^{℘}\frac{{\left(℘-\phi \right)}^{\zeta -1}{\underline{\vartheta }}_{r}\left(\phi \right)}{\Gamma (\zeta )}d\phi ,\;\zeta ,\phi \in \left(0,\infty \right),\\ & I^{\zeta }{\bar{\vartheta }}_{r}\left(℘\right)=\int_{0}^{℘}\frac{{\left(℘-\phi \right)}^{\zeta -1}{\bar{\vartheta }}_{r}\left(\phi \right)}{\Gamma (\zeta )}d\phi ,\;\zeta ,\phi \in \left(0,\infty \right). \end{array} \end{array}$$

**Lemma 2.4** Let $\tilde{\vartheta }\in C^{F}\left[0,b\right]\cap L^{F}\left[0,b\right]$ so that $[{\underline{\vartheta }}_{r}\left(℘\right),{\bar{\vartheta }}_{r}\left(℘\right)],r\in \left[0,1\right]$ and **℘**_0_ ∈ (0, *b*), thus, the fuzzy Caputo fractional derivative is expressed as

$$\begin{array}{c} {\left[D^{\zeta }\tilde{\vartheta }({℘}_{0})\right]}_{r}=[D^{\zeta }{\underline{\vartheta }}_{r}({℘}_{0}),D^{\zeta }{\bar{\vartheta }}_{r}({℘}_{0})],0<\zeta \leq 1, \end{array}$$

where

$$\begin{array}{c} \begin{array}{cc} & D^{\zeta }{\underline{\vartheta }}_{r}({℘}_{0})={\left[\int_{0}^{℘}\frac{{\left(℘-\phi \right)}^{m-\zeta -1}\frac{d^{mm}}{d\phi^{m}}\vartheta_{r}\left(\phi \right)}{\Gamma (m-\zeta )}d\phi \right]}_{℘={℘}_{0}},\\ & D^{\zeta }{\bar{\vartheta }}_{r}({℘}_{0})={\left[\int_{0}^{℘}\frac{{\left(℘-\phi \right)}^{m-\zeta -1}\frac{d^{m}}{d\phi^{m}}{\bar{\vartheta }}_{r}\left(\phi \right)}{\Gamma (m-\zeta )}d\phi \right]}_{℘={℘}_{0}}, \end{array} \end{array}$$

such that the integral on the right side converges and *m* = ⌈*ζ*⌉. Since *ζ* ∈ (0, 1] so *m* = 1.

## 3 Basic ideas of the HPS

In this section, we demonstrate the idea of HPS where the solutions are derived in terms of series solution. Consider the following general problem

$$\begin{array}{c} L\left(\vartheta \right)-f_{1}\left(r\right)=0,\;r\in D, \end{array}$$ (3)

with conditions

$$\begin{array}{c} M(\vartheta ,\frac{\partial \vartheta }{\partial r})=0,\;r\in \Gamma , \end{array}$$ (4)

where *L* and *M* are expressed as a general function and boundary operator respectively, *f*_1_(*r*) is known parameter and Γ as a interval of the domain *D*. Now, if we split *L* into two operators such that *A*_1_ and *A*_2_ are identified as linear and nonlinear operators respectively, then Eq (2) follows as

$$\begin{array}{c} A_{1}\left(\vartheta \right)+A_{2}\left(\vartheta \right)-f_{1}\left(r\right)=0. \end{array}$$ (5)

Let ϑ(r,p):D×[0,1]→R, such that

$$\begin{array}{c} H\left(\vartheta ,p\right)=\left(1-p\right)\left[A_{1}\left(\vartheta \right)-A_{1}\left(\vartheta_{0}\right)\right]+p\left[A_{1}\left(\vartheta \right)-A_{2}\left(\vartheta \right)-f_{1}\left(r\right)\right], \end{array}$$

or

$$\begin{array}{c} H\left(\vartheta ,p\right)=A_{1}\left(\vartheta \right)-A_{1}\left(\vartheta_{0}\right)+pL\left(\vartheta_{0}\right)+p\left[A_{2}\left(\vartheta \right)-f_{1}\left(r\right)\right]=0, \end{array}$$

where *ϑ*_0_ is starting point of relation (3) that completes the boundary conditions, and *p* ∈ [0, 1] is homotopy element. The above equations may also be written as

$$\begin{array}{c} \begin{array}{cc} H(\vartheta ,0) & =A_{1}\left(\vartheta \right)-A_{1}\left(\vartheta_{0}\right)=0,\\ H(\vartheta ,0) & =A\left(\vartheta \right)-f_{1}\left(r\right)=0. \end{array} \end{array}$$ (6)

The function *ϑ*(*r*, *p*) transforms *ϑ*_0_(*r*) to *ϑ*(*r*) due to the rising value of *p* from zero to one. In topology, this is known as deformation, where *A*_1_(*ϑ*) − *A*_1_(*ϑ*_0_) and *A*(*ϑ*) − *f*_1_(*r*) are expressed as homotopic. As *p* ∈ [0, 1] is a basic number, so that we can handle the solution of Eq (3) in the form of power series such that

$$\begin{array}{cc} \vartheta & =\vartheta_{0}+p\vartheta_{1}+p^{2}\vartheta_{2}+p^{3}\vartheta_{3}+\cdots =\sum_{i=0}^{\infty }\;p^{i}\vartheta_{i}. \end{array}$$ (7)

Let *p* = 1, the above Eq (7) yields as

$$\begin{array}{c} \vartheta =\underset{p\to 1}{\text{lim}}\;\vartheta =\vartheta_{0}+\vartheta_{1}+\vartheta_{2}+\vartheta_{3}+\cdots =\sum_{i=0}^{\infty }\vartheta_{i}. \end{array}$$ (8)

## 4 Development of SHPTS

In this segment, we propose the concept of SHPTS for the analytical results of fuzzy fractional two dimensional heat problem. Our proposed strategy demonstrates that there is no requirement of assumption and restriction of variables during the development. In this work, we consider the fractional order *α* for the lower bound solution and the fractional order *β* for the upper bound solution.

### 4.1 Methodology for lower bound solution

We encounter a fractional differential problem of order *α* in lower bound form

$$\begin{array}{c} D_{℘}^{\alpha }\underline{\vartheta }\left(ℑ,\varsigma ,℘\right)=L_{1}\underline{\vartheta }\left(ℑ,\varsigma ,℘\right)+L_{2}\underline{\vartheta }\left(ℑ,\varsigma ,℘\right)+g\left(ℑ,\varsigma ,℘\right), \end{array}$$ (9)

with initial condition

$$\begin{array}{c} \underline{\vartheta }\left(ℑ,\varsigma ,0\right)=f\left(ℑ,\varsigma \right), \end{array}$$ (10)

Using ST on Eq (9), we obtain

$$\begin{array}{c} S\left[D_{℘}^{\alpha }\underline{\vartheta }\left(ℑ,\varsigma ,℘\right)\right]=S\left[L_{1}\underline{\vartheta }\left(ℑ,\varsigma ,℘\right)+L_{2}\underline{\vartheta }\left(ℑ,\varsigma ,℘\right)+g\left(ℑ,\varsigma ,℘\right)\right]. \end{array}$$

The Sawi transform in fractional derivative is used as

$$\begin{array}{c} \frac{1}{\theta^{\alpha }}S\left[\underline{\vartheta }\left(ℑ,\varsigma ,℘\right)\right]-\frac{1}{\theta^{\alpha +1}}\underline{\vartheta }\left(ℑ,0\right)=S\left[L_{1}\underline{\vartheta }\left(ℑ,\varsigma ,℘\right)+L_{2}\underline{\vartheta }\left(ℑ,\varsigma ,℘\right)+g\left(ℑ,\varsigma ,℘\right)\right]. \end{array}$$

We can solve it as

$$\begin{array}{c} S\left[\underline{\vartheta }\left(ℑ,\varsigma ,℘\right)\right]=\frac{1}{\theta }f\left(ℑ,\varsigma \right)+\theta^{\alpha }S[L_{1}\underline{\vartheta }\left(ℑ,\varsigma ,℘\right)+L_{2}\underline{\vartheta }\left(ℑ,\varsigma ,℘\right)+g\left(ℑ,\varsigma ,℘\right)]. \end{array}$$

Operating inverse ST on above equation, we obtain

$$\begin{array}{c} \underline{\vartheta }\left(ℑ,\varsigma ,℘\right)=Q\left(ℑ,\varsigma ,℘\right)+S^{-1}[\theta^{\alpha }S\{L_{1}\underline{\vartheta }\left(ℑ,\varsigma ,℘\right)+L_{2}\underline{\vartheta }\left(ℑ,\varsigma ,℘\right)\}], \end{array}$$ (11)

where

$$\begin{array}{c} Q\left(ℑ,\varsigma ,℘\right)=S^{-1}[\frac{1}{\theta }f\left(ℑ,\varsigma \right)+\theta^{\alpha }S\left[g\left(ℑ,\varsigma ,℘\right)\right]]. \end{array}$$

Now, HPS yields as

$$\begin{array}{c} \underline{\vartheta }\left(ℑ,\varsigma ,℘\right)=\sum_{i=0}^{\infty }p^{i}{\underline{\vartheta }}_{i}\left(ℑ,\varsigma ,℘\right), \end{array}$$ (12)

and

$$\begin{array}{c} L_{2}\underline{\vartheta }\left(ℑ,\varsigma ,℘\right)=\sum_{i=0}^{\infty }p^{i}H_{i}\left(\underline{\vartheta }\right). \end{array}$$ (13)

The components of *H*_*i*_ are defined as

$$\begin{array}{c} H_{n}\left({\underline{\vartheta }}_{0},{\underline{\vartheta }}_{1},\cdots ,{\underline{\vartheta }}_{n}\right)=\frac{1}{n!}\frac{\partial^{n}}{\partial p^{n}}{\left(L_{2}(\sum_{i=0}^{\infty }p^{i}{\underline{\vartheta }}_{i})\right)}_{p=0},\;\;\;\;n=0,1,2,\cdots \end{array}$$

Putting Eqs (12) and (13) into Eq (11), we get

$$\begin{array}{c} \sum_{i=0}^{\infty }p^{i}{\underline{\vartheta }}_{i}\left(ℑ,\varsigma ,℘\right)=G\left(ℑ,\varsigma ,℘\right)+S-1[\theta^{\alpha }\varsigma \{L_{1}\sum_{i=0}^{\infty }p^{i}{\underline{\vartheta }}_{i}\left(ℑ,\varsigma ,℘\right)+\sum_{i=0}^{\infty }p^{i}H_{i}\left(\underline{\vartheta }\right)\}]. \end{array}$$ (14)

By examining the related factors of *p*, we arrive at

$$\begin{array}{cc} p^{0} & :\underline{\vartheta_{0}}\left(ℑ,\varsigma ,℘\right)=G\left(ℑ,\varsigma ,℘\right),\\ p^{1} & :\underline{\vartheta_{1}}\left(ℑ,\varsigma ,℘\right)=S^{-1}[\theta^{\alpha }S\{\underline{\vartheta_{0}}\left(ℑ,\varsigma ,℘\right)+H_{0}\left(\underline{\vartheta }\right)\}],\\ p^{2} & :\underline{\vartheta_{2}}\left(ℑ,\varsigma ,℘\right)=S^{-1}[\theta^{\alpha }S\{\underline{\vartheta_{1}}\left(ℑ,\varsigma ,℘\right)+H_{1}\left(\underline{\vartheta }\right)\}],\\ p^{3} & :\underline{\vartheta_{3}}\left(ℑ,\varsigma ,℘\right)=S^{-1}[\theta^{\alpha }S\{\underline{\vartheta_{2}}\left(ℑ,\varsigma ,℘\right)+H_{2}\left(\underline{\vartheta }\right)\}],\\ & \vdots . \end{array}$$

In other words

$$\begin{array}{c} \underline{\vartheta }\left(ℑ,\varsigma ,℘\right)={\underline{\vartheta }}_{0}\left(ℑ,\varsigma ,℘\right)+{\underline{\vartheta }}_{1}\left(ℑ,\varsigma ,℘\right)+{\underline{\vartheta }}_{2}\left(ℑ,\varsigma ,℘\right)+\cdots . \end{array}$$ (15)

### 4.2 Methodology for upper bound solution

We encounter a fractional differential problem of order *β* in upper bound form

$$\begin{array}{c} D_{℘}^{\beta }\bar{\vartheta }\left(ℑ,\varsigma ,℘\right)=L_{1}\bar{\vartheta }\left(ℑ,\varsigma ,℘\right)+L_{2}\bar{\vartheta }\left(ℑ,\varsigma ,℘\right)+g\left(ℑ,℘\right), \end{array}$$ (16)

subjected to the condition

$$\begin{array}{c} \bar{\vartheta }\left(ℑ,\varsigma ,0\right)=f\left(ℑ,\varsigma ,0\right), \end{array}$$ (17)

Employing ST on Eq (16), we get

$$\begin{array}{c} S\left[D_{℘}^{\beta }\bar{\vartheta }\left(ℑ,\varsigma ,℘\right)\right]=S\left[L_{1}\bar{\vartheta }\left(ℑ,\varsigma ,℘\right)+L_{2}\bar{\vartheta }\left(ℑ,\varsigma ,℘\right)+g\left(ℑ,\varsigma ,℘\right)\right]. \end{array}$$

The Sawi transform in fractional derivative is used as

$$\begin{array}{c} \frac{1}{\theta^{\beta }}S\left[\bar{\vartheta }\left(ℑ,\varsigma ,℘\right)\right]-\frac{1}{\theta^{\beta +1}}\bar{\vartheta }\left(ℑ,\varsigma ,0\right)=S\left[L_{1}\bar{\vartheta }\left(ℑ,\varsigma ,℘\right)+L_{2}\bar{\vartheta }\left(ℑ,\varsigma ,℘\right)+g\left(ℑ,\varsigma ,℘\right)\right]. \end{array}$$

We can solve it as

$$\begin{array}{c} S\left[\bar{\vartheta }\left(ℑ,\varsigma ,℘\right)\right]=\frac{1}{\theta }f\left(ℑ,\varsigma ,0\right)+\theta^{\beta }S[L_{1}\bar{\vartheta }\left(ℑ,\varsigma ,℘\right)+L_{2}\bar{\vartheta }\left(ℑ,\varsigma ,℘\right)+g\left(ℑ,\varsigma ,℘\right)]. \end{array}$$

Operating inverse ST on above equation, we obtain

$$\begin{array}{c} \bar{\vartheta }\left(ℑ,\varsigma ,℘\right)=G\left(ℑ,\varsigma ,℘\right)+S^{-1}[\theta^{\beta }S\{L_{1}\bar{\vartheta }\left(ℑ,\varsigma ,℘\right)+L_{2}\bar{\vartheta }\left(ℑ,\varsigma ,℘\right)\}], \end{array}$$ (18)

where

$$\begin{array}{c} G\left(ℑ,\varsigma ,℘\right)=S^{-1}[\frac{1}{\theta }f\left(ℑ,\varsigma ,0\right)+\theta^{\beta }S\left[g\left(ℑ,\varsigma ,℘\right)\right]]. \end{array}$$

Now, HPS yields as

$$\begin{array}{c} \bar{\vartheta }\left(ℑ,\varsigma ,℘\right)=\sum_{i=0}^{\infty }\;p^{i}{\bar{\vartheta }}_{i}\left(ℑ,\varsigma ,℘\right), \end{array}$$ (19)

and

$$\begin{array}{c} L_{2}\bar{\vartheta }\left(ℑ,\varsigma ,℘\right)=\sum_{i=0}^{\infty }\;p^{i}H_{i}\left(\bar{\vartheta }\right). \end{array}$$ (20)

The components of *H*_*i*_ are defined as

$$\begin{array}{c} H_{n}\left({\bar{\vartheta }}_{0},{\bar{\vartheta }}_{1},\cdots ,{\bar{\vartheta }}_{n}\right)=\frac{1}{n!}\frac{\partial^{n}}{\partial p^{n}}{\left(L_{2}(\sum_{i=0}^{\infty }\;p^{i}{\bar{\vartheta }}_{i})\right)}_{p=0},\;\;\;\;n=0,1,2,\cdots \end{array}$$

Putting Eqs (19) and (20) into Eq (18), we get

$$\begin{array}{c} \sum_{i=0}^{\infty }\;p^{i}{\bar{\vartheta }}_{i}\left(ℑ,\varsigma ,℘\right)=G\left(ℑ,\varsigma ,℘\right)+S-1[\theta^{\beta }S\{L_{1}\sum_{i=0}^{\infty }\;p^{i}{\bar{\vartheta }}_{i}\left(ℑ,\varsigma ,℘\right)+\sum_{i=0}^{\infty }p^{i}H_{i}\left(\bar{\vartheta }\right)\}]. \end{array}$$ (21)

By examining the related factors of *p*, we arrive at

$$\begin{array}{cc} p^{0} & :{\bar{\vartheta }}_{0}\left(ℑ,\varsigma ,℘\right)=G\left(ℑ,\varsigma ,℘\right),\\ p^{1} & :{\bar{\vartheta }}_{1}\left(ℑ,\varsigma ,℘\right)=S^{-1}[\theta^{\beta }S\{{\bar{\vartheta }}_{0}\left(ℑ,\varsigma ,℘\right)+H_{0}\left(\bar{\vartheta }\right)\}],\\ p^{2} & :{\bar{\vartheta }}_{2}\left(ℑ,\varsigma ,℘\right)=S^{-1}[\theta^{\beta }S\{{\bar{\vartheta }}_{1}\left(ℑ,\varsigma ,℘\right)+H_{1}\left(\bar{\vartheta }\right)\}],\\ p^{3} & :{\bar{\vartheta }}_{3}\left(ℑ,\varsigma ,℘\right)=S^{-1}[\theta^{\beta }S\{{\bar{\vartheta }}_{2}\left(ℑ,\varsigma ,℘\right)+H_{2}\left(\bar{\vartheta }\right)\}],\\ & \vdots . \end{array}$$

In other words

$$\begin{array}{c} \bar{\vartheta }\left(ℑ,\varsigma ,℘\right)={\bar{\vartheta }}_{0}\left(ℑ,\varsigma ,℘\right)+{\bar{\vartheta }}_{1}\left(ℑ,\varsigma ,℘\right)+{\bar{\vartheta }}_{2}\left(ℑ,\varsigma ,℘\right)+\cdots . \end{array}$$ (22)

## 5 Convergence analysis

**Theorem 5.1** Suppose [*a*, *b*] × [0, *T*] be the rectangular interval that establishes the Banach space *B* ≡ *C*([*a*, *b*] × [0, *T*]). Then, Eq (22) $\vartheta \left(ℑ,\varsigma ,℘\right)=\sum_{i=0}^{\infty }\vartheta_{i}\left(ℑ,\varsigma ,℘\right)$ is continuous if *ϑ*_0_ ∈ *B* is bounded where ‖*ϑ*_*i*+1_‖ ≤ ‖*ϑ*_*i*_‖, ∀*ϑ*_*i*_ ∈ *B* with 0 < *μ* < 1.

**Proof:** Using a series $\left\{F_{r}\right\}$ as a partial result of Eq (22), we get

$$\begin{array}{c} \begin{array}{cc} F_{0} & =\vartheta_{0}\left(ℑ,\varsigma ,℘\right),\\ F_{1} & =\vartheta_{0}\left(ℑ,\varsigma ,℘\right)+\vartheta_{1}\left(ℑ,\varsigma ,℘\right),\\ F_{2} & =\vartheta_{0}\left(ℑ,\varsigma ,℘\right)+\vartheta_{1}\left(ℑ,\varsigma ,℘\right)+\vartheta_{2}\left(ℑ,\varsigma ,℘\right),\\ & \vdots \\ F_{r} & =\vartheta_{0}\left(ℑ,\varsigma ,℘\right)+\vartheta_{1}\left(ℑ,\varsigma ,℘\right)+\vartheta_{2}\left(ℑ,\varsigma ,℘\right)+\ldots +\vartheta_{r}\left(ℑ,\varsigma ,℘\right). \end{array} \end{array}$$ (23)

We then show ${\left\{F_{r}\right\}}_{r=0}^{\infty }$ is a Cauchy sequence in *B* so that this theorem can be verified. Thus,

$$\begin{array}{c} \begin{array}{cc} ∥F_{r+1}-F_{r}∥ & =∥\vartheta_{r+1}\left(ℑ,\varsigma ,℘\right)∥,\\ & \leq \mu ∥\vartheta_{r}\left(ℑ,\varsigma ,℘\right)∥,\\ & \leq \mu^{2}∥\vartheta_{r-1}\left(ℑ,\varsigma ,℘\right)∥,\\ & \vdots \\ & \leq \mu^{r+1}∥\vartheta_{0}\left(ℑ,\varsigma ,℘\right)∥. \end{array} \end{array}$$ (24)

Thus, for every pair *r*, *n* ∈ *N* with *r* > *n*, there is

$$\begin{array}{c} \begin{array}{cc} ∥F_{r}-F_{n}∥ & =∥(F_{r}-F_{r-1})+(F_{r-1}-F_{r-2})+(F_{r-2}-F_{r-3})+\ldots +(F_{n+1}-F_{n})∥,\\ & \leq ∥F_{r}-F_{r-1}∥+∥F_{r-1}-F_{r-2}∥+∥F_{r-2}-F_{r-3}∥+\ldots +∥F_{n+1}-F_{n}∥,\\ & \leq \mu^{r}∥\vartheta_{0}\left(ℑ,\varsigma ,℘\right)∥+\mu^{r-1}∥\vartheta_{0}\left(ℑ,\varsigma ,℘\right)∥+\ldots +\mu^{n+1}∥\vartheta_{0}\left(ℑ,\varsigma ,℘\right)∥,\\ & \leq \beta ∥\vartheta_{0}\left(ℑ,\varsigma ,℘\right)∥. \end{array} \end{array}$$ (25)

in which $\beta =\frac{\left(1-\mu^{r-n}\right)}{\left(1-\mu \right)}\mu^{n+1}$. Being that $\vartheta_{0}\left(ℑ,\varsigma ,℘\right)$ is continuous, so $∥\vartheta_{0}\left(ℑ,\varsigma ,℘\right)∥<\infty$. Since *n* increases and *n* → ∞ tends to *β* → 0 over 0 < *μ* < 1, hence

$$\begin{array}{c} \underset{n\to \infty r\to \infty }{\text{lim}}∥F_{r}-F_{n}∥=0. \end{array}$$ (26)

This means ${\left\{F_{r}\right\}}_{r=0}^{\infty }$ shows a Cauchy sequence in *B*. Therefore, the series solution of Eq (22) is convergent.

**Theorem 5.2** Let $\sum_{k=0}^{n}\vartheta_{k}\left(ℑ,\varsigma ,℘\right)$ shows the approximation of Eq (16), ultimately the absolute error is identified as

$$\begin{array}{c} ∥\vartheta \left(ℑ,\varsigma ,℘\right)-\sum_{k=0}^{n}\vartheta_{k}\left(ℑ,\varsigma ,℘\right)∥\leq \frac{\mu^{n+1}}{1-\mu }∥\vartheta_{0}\left(ℑ,\varsigma ,℘\right)∥, \end{array}$$ (27)

where *μ* shows a numeric number such that $\frac{∥\vartheta_{i+1}∥}{∥\vartheta_{i}∥}\leq \mu$.

**Proof:** Applying Theorem (5.1) to Eq (25), we get

$$\begin{array}{c} ∥F_{r}-F_{n}∥\leq \beta ∥\vartheta_{0}\left(ℑ,\varsigma ,℘\right)∥, \end{array}$$ (28)

where

$$\begin{array}{c} \beta =\frac{\left(1-\mu^{r-n}\right)}{\left(1-\mu \right)}\mu^{n+1}. \end{array}$$

Since ${\left\{F_{r}\right\}}_{r=0}^{\infty }\to \vartheta \left(ℑ,\varsigma ,℘\right)$ as *r* → ∞ and from Eq (23), we obtain $F_{n}=\sum_{k=0}^{n}\vartheta_{k}\left(ℑ,\varsigma ,℘\right)$,

$$\begin{array}{c} ∥\vartheta \left(ℑ,\varsigma ,℘\right)-\sum_{k=0}^{n}\vartheta_{k}\left(ℑ,\varsigma ,℘\right)∥\leq \beta ∥\vartheta_{0}\left(ℑ,\varsigma ,℘\right)∥, \end{array}$$ (29)

where (1 − *μ*^*r*−*n*^) < 1 and 0 < *μ* < 1

$$\begin{array}{c} ∥\vartheta \left(ℑ,\varsigma ,℘\right)-\sum_{k=0}^{n}\vartheta_{k}\left(ℑ,\varsigma ,℘\right)∥\leq \frac{\mu^{n+1}}{1-\mu }∥\vartheta_{0}\left(ℑ,\varsigma ,℘\right)∥. \end{array}$$ (30)

Thus, the truth is proof.

## 6 Applications

In this section, we put our suggested technique into practice for the analytical results of a heat problem in two-dimensional fuzzy fractional form with lower and upper-bound solutions. We analyze the findings in terms of a series that quickly converges. The surface and contour plots are displayed to show the efficiency of suggested scheme. The results show that this approachis relatively simple to implement for fractional order fuzzy problems.

### 6.1 Example 1

Consider the 2D homogeneous time-fractional heat flow problem

$$\begin{array}{c} D_{℘}^{\alpha ,\;\beta }\tilde{\vartheta }\left(ℑ,\varsigma ,℘\right)={\tilde{\vartheta }}_{ℑℑ}\left(ℑ,\varsigma ,℘\right)+{\tilde{\vartheta }}_{\varsigma \varsigma }\left(ℑ,\varsigma ,℘\right)+ℑ+\varsigma +℘, \end{array}$$ (31)

with the initial condition

$$\begin{array}{c} \tilde{\vartheta }\left(ℑ,\varsigma ,0\right)=\tilde{k}e^{-(ℑ+\varsigma )}. \end{array}$$ (32)

where $\tilde{k}=\left[\underline{k},\bar{k}\right]=\left[r-1,1-r\right]$.

#### 6.1.1 For lower bound solution

Since, we have

$$\begin{array}{c} \frac{\partial^{\alpha }\underline{\vartheta }}{\partial {℘}^{\alpha }}=\frac{\partial^{2}\underline{\vartheta }}{\partial {ℑ}^{2}}+\frac{\partial^{2}\underline{\vartheta }}{\partial \varsigma^{2}}+ℑ+\varsigma +℘, \end{array}$$ (33)

subjected to the condition

$$\begin{array}{c} \underline{\vartheta }\left(ℑ,\varsigma ,0\right)=\underline{k}e^{-(ℑ+\varsigma )}. \end{array}$$ (34)

where $\underline{k}=r-1$.

Apply ST on Eq (33), we get

$$\begin{array}{c} S[\frac{\partial^{\alpha }\underline{\vartheta }}{\partial {℘}^{\alpha }}]=S[\frac{\partial^{2}\underline{\vartheta }}{\partial {ℑ}^{2}}+\frac{\partial^{2}\underline{\vartheta }}{\partial \varsigma^{2}}+ℑ+\varsigma +℘]. \end{array}$$

Using the Sawi transform in fractional derivative, we obtain

$$\begin{array}{c} S\left[\underline{\vartheta }\left(ℑ,\varsigma ,℘\right)\right]=\frac{1}{\theta }\underline{\vartheta }\left(ℑ,\varsigma ,0\right)+\theta^{\alpha }S[\frac{\partial^{2}\underline{\vartheta }}{\partial {ℑ}^{2}}+\frac{\partial^{2}\underline{\vartheta }}{\partial \varsigma^{2}}+ℑ+\varsigma +℘]. \end{array}$$

In other way, we can also write it as

$$\begin{array}{c} S\left[\underline{\vartheta }\left(ℑ,\varsigma ,℘\right)\right]=\frac{1}{\theta }\underline{\vartheta }\left(ℑ,\varsigma ,0\right)+\theta^{\alpha }S\left[ℑ+\varsigma +℘\right]+\theta^{\alpha }S[\frac{\partial^{2}\underline{\vartheta }}{\partial {ℑ}^{2}}+\frac{\partial^{2}\underline{\vartheta }}{\partial \varsigma^{2}}]. \end{array}$$

Using the inverse ST, we get

$$\begin{array}{c} \vartheta \left(ℑ,\varsigma ,℘\right)=\underline{\vartheta }\left(ℑ,\varsigma ,0\right)+ℑ\frac{{℘}^{\alpha }}{\alpha !}+\varsigma \frac{{℘}^{\alpha }}{\alpha !}+\frac{{℘}^{\alpha +1}}{(\alpha +1)!}+S^{-1}[\theta^{\alpha }S[\frac{\partial^{2}\underline{\vartheta }}{\partial {ℑ}^{2}}+\frac{\partial^{2}\underline{\vartheta }}{\partial \varsigma^{2}}]. \end{array}$$ (35)

Implement the idea of HPS on Eq (35), we obtain the He’s iterations such as

$$\begin{array}{c} \sum_{i=0}^{\infty }p^{i}\underline{\vartheta }\left(ℑ,\varsigma ,℘\right)=\underline{\vartheta }\left(ℑ,\varsigma ,0\right)+ℑ\frac{{℘}^{\alpha }}{\alpha !}+\varsigma \frac{{℘}^{\alpha }}{\alpha !}+\frac{{℘}^{\alpha +1}}{(\alpha +1)!}+S^{-1}[\theta^{\alpha }S[\sum_{i=0}^{\infty }p^{i}\frac{\partial^{2}\underline{\vartheta_{i}}}{\partial {ℑ}^{2}}+\sum_{i=0}^{\infty }p^{i}\frac{\partial^{2}\underline{\vartheta_{i}}}{\partial \varsigma^{2}}]. \end{array}$$

By examining the related factors of *p*, we arrive at

$$\begin{array}{cc} p^{0} & :\underline{\vartheta_{0}}\left(ℑ,\varsigma ,℘\right)=\underline{\vartheta }\left(ℑ,\varsigma ,0\right)=\underline{k}e^{-(ℑ+\varsigma )}+ℑ\frac{{℘}^{\alpha }}{\alpha !}+\varsigma \frac{{℘}^{\alpha }}{\alpha !}+\frac{{℘}^{\alpha +1}}{(\alpha +1)!},\\ p^{1} & :\underline{\vartheta_{1}}\left(ℑ,\varsigma ,℘\right)=S^{-1}[\theta^{\alpha }S\{\frac{\partial^{2}\underline{\vartheta_{0}}}{\partial {ℑ}^{2}}+\frac{\partial^{2}\underline{\vartheta_{0}}}{\partial \varsigma^{2}}\}]=2\underline{k}e^{-(ℑ+\varsigma )}\frac{{℘}^{\alpha }}{\alpha !},\\ p^{2} & :\underline{\vartheta_{2}}\left(ℑ,\varsigma ,℘\right)=S^{-1}[\theta^{\alpha }S\{\frac{\partial^{2}\underline{\vartheta_{1}}}{\partial {ℑ}^{2}}+\frac{\partial^{2}\underline{\vartheta_{1}}}{\partial \varsigma^{2}}\}]=4\underline{k}e^{-(ℑ+\varsigma )}\frac{{℘}^{2\alpha }}{(2\alpha )!},\\ p^{3} & :\underline{\vartheta_{3}}\left(ℑ,\varsigma ,℘\right)=S^{-1}[\theta^{\alpha }S\{\frac{\partial^{2}\underline{\vartheta_{2}}}{\partial {ℑ}^{2}}+\frac{\partial^{2}\underline{\vartheta_{2}}}{\partial \varsigma^{2}}\}]=8\underline{k}e^{-(ℑ+\varsigma )}\frac{{℘}^{3\alpha }}{(3\alpha )!},\\ & \vdots . \end{array}$$

In other words

$$\begin{array}{c} \begin{array}{cc} \underline{\vartheta }\left(ℑ,\varsigma ,℘\right) & =\underline{\vartheta_{0}}\left(ℑ,\varsigma ,℘\right)+\underline{\vartheta_{1}}\left(ℑ,\varsigma ,℘\right)+\underline{\vartheta_{2}}\left(ℑ,\varsigma ,℘\right)+\underline{\vartheta_{3}}\left(ℑ,\varsigma ,℘\right)+\cdots ,\\ & =\underline{k}e^{-(ℑ+\varsigma )}+ℑ\frac{{℘}^{\alpha }}{\alpha !}+\varsigma \frac{{℘}^{\alpha }}{\alpha !}+\frac{{℘}^{\alpha +1}}{(\alpha +1)!}+2\underline{k}e^{-(ℑ+\varsigma )}\frac{{℘}^{\alpha }}{\alpha !}+4\underline{k}e^{-(ℑ+\varsigma )}\frac{{℘}^{2\alpha }}{(2\alpha )!}+8\underline{k}e^{-(ℑ+\varsigma )}\frac{{℘}^{3\alpha }}{(3\alpha )!}+\cdots . \end{array} \end{array}$$ (36)

**Remark:** If g(ℑ,ς,℘)=0, then above equation becomes as

$$\begin{array}{c} \underline{\vartheta }\left(ℑ,\varsigma ,℘\right)=\underline{k}e^{-(ℑ+\varsigma )}+2\underline{k}e^{-(ℑ+\varsigma )}\frac{{℘}^{\alpha }}{\alpha !}+4\underline{k}e^{-(ℑ+\varsigma )}\frac{{℘}^{2\alpha }}{(2\alpha )!}+8\underline{k}e^{-(ℑ+\varsigma )}\frac{{℘}^{3\alpha }}{(3\alpha )!}+\cdots . \end{array}$$ (37)

which can be closed form

$$\begin{array}{c} \underline{\vartheta }\left(ℑ,\varsigma ,℘\right)=\underline{k}e^{-(ℑ+\varsigma )}\sum_{n=0}^{\infty }\frac{{\left(2{℘}^{\alpha }\right)}^{n}}{(n\alpha )!}. \end{array}$$ (38)

#### 6.1.2 For upper bound solution

Since, we have

$$\begin{array}{c} \frac{\partial^{\beta }\bar{\vartheta }}{\partial {℘}^{\beta }}=\frac{\partial^{2}\bar{\vartheta }}{\partial {ℑ}^{2}}+\frac{\partial^{2}\bar{\vartheta }}{\partial \varsigma^{2}}+ℑ+\varsigma +℘, \end{array}$$ (39)

with the initial condition

$$\begin{array}{c} \bar{\vartheta }\left(ℑ,\varsigma ,0\right)=\bar{k}e^{-(ℑ+\varsigma )}. \end{array}$$ (40)

where $\bar{k}=1-r$.

Apply ST on Eq (39), we get

$$\begin{array}{c} S[\frac{\partial^{\beta }\bar{\vartheta }}{\partial {℘}^{\beta }}]=S[\frac{\partial^{2}\bar{\vartheta }}{\partial {ℑ}^{2}}+\frac{\partial^{2}\bar{\vartheta }}{\partial \varsigma^{2}}+ℑ+\varsigma +℘]. \end{array}$$

Using the Sawi transform in fractional derivative, we obtain

$$\begin{array}{c} S\left[\bar{\vartheta }\left(ℑ,\varsigma ,℘\right)\right]=\frac{1}{\theta }\bar{\vartheta }\left(ℑ,\varsigma ,0\right)+\theta^{\beta }S[\frac{\partial^{2}\bar{\vartheta }}{\partial {ℑ}^{2}}+\frac{\partial^{2}\bar{\vartheta }}{\partial \varsigma^{2}}+ℑ+\varsigma +℘]. \end{array}$$

In other way, we can also write it as

$$\begin{array}{c} S\left[\bar{\vartheta }\left(ℑ,\varsigma ,℘\right)\right]=\frac{1}{\theta }\bar{\vartheta }\left(ℑ,\varsigma ,0\right)+\theta^{\beta }S\left[ℑ+\varsigma +℘\right]+\theta^{\beta }S[\frac{\partial^{2}\bar{\vartheta }}{\partial {ℑ}^{2}}+\frac{\partial^{2}\bar{\vartheta }}{\partial \varsigma^{2}}]. \end{array}$$

Using inverse ST, we get

$$\begin{array}{c} \vartheta \left(ℑ,\varsigma ,℘\right)=\bar{\vartheta }\left(ℑ,\varsigma ,0\right)+ℑ\frac{{℘}^{\beta }}{\beta !}+\varsigma \frac{{℘}^{\beta }}{\beta !}+\frac{{℘}^{\beta +1}}{(\beta +1)!}+S^{-1}[\theta^{\beta }S[\frac{\partial^{2}\bar{\vartheta }}{\partial {ℑ}^{2}}+\frac{\partial^{2}\bar{\vartheta }}{\partial \varsigma^{2}}]. \end{array}$$ (41)

Implement the idea of HPS on Eq (41), we obtain the He’s iterations such as

$$\begin{array}{c} \sum_{i=0}^{\infty }p^{i}\bar{\vartheta }\left(ℑ,\varsigma ,℘\right)=\bar{\vartheta }\left(ℑ,\varsigma ,0\right)+ℑ\frac{{℘}^{\beta }}{\beta !}+\varsigma \frac{{℘}^{\beta }}{\beta !}+\frac{{℘}^{\beta +1}}{(\beta +1)!}+S^{-1}[\theta^{\beta }S[\sum_{i=0}^{\infty }\;p^{i}\frac{\partial^{2}\bar{\vartheta_{i}}}{\partial {ℑ}^{2}}+\sum_{i=0}^{\infty }\;p^{i}\frac{\partial^{2}\bar{\vartheta_{i}}}{\partial \varsigma^{2}}]. \end{array}$$

By comparing the related factors of *p*, we obtain

$$\begin{array}{cc} p^{0} & :\bar{\vartheta_{0}}\left(ℑ,\varsigma ,℘\right)=\bar{\vartheta }\left(ℑ,\varsigma ,0\right)=\bar{k}e^{-(ℑ+\varsigma )}+ℑ\frac{{℘}^{\beta }}{\beta !}+\varsigma \frac{{℘}^{\beta }}{\beta !}+\frac{{℘}^{\beta +1}}{(\beta +1)!},\\ p^{1} & :\bar{\vartheta_{1}}\left(ℑ,\varsigma ,℘\right)=S^{-1}[\theta^{\beta }S\{\frac{\partial^{2}\bar{\vartheta_{0}}}{\partial {ℑ}^{2}}+\frac{\partial^{2}\bar{\vartheta_{0}}}{\partial \varsigma^{2}}\}]=2\bar{k}e^{-(ℑ+\varsigma )}\frac{{℘}^{\beta }}{\beta !},\\ p^{2} & :\bar{\vartheta_{2}}\left(ℑ,\varsigma ,℘\right)=S^{-1}[\theta^{\beta }S\{\frac{\partial^{2}\bar{\vartheta_{1}}}{\partial {ℑ}^{2}}+\frac{\partial^{2}\bar{\vartheta_{1}}}{\partial \varsigma^{2}}\}]=4\bar{k}e^{-(ℑ+\varsigma )}\frac{{℘}^{2\beta }}{(2\beta )!},\\ p^{3} & :\bar{\vartheta_{3}}\left(ℑ,\varsigma ,℘\right)=S^{-1}[\theta^{\beta }S\{\frac{\partial^{2}\bar{\vartheta_{2}}}{\partial {ℑ}^{2}}+\frac{\partial^{2}\bar{\vartheta_{2}}}{\partial \varsigma^{2}}\}]=8\bar{k}e^{-(ℑ+\varsigma )}\frac{{℘}^{3\beta }}{(3\beta )!},\\ & \vdots . \end{array}$$

In other words

$$\begin{array}{c} \begin{array}{cc} \bar{\vartheta }\left(ℑ,\varsigma ,℘\right) & =\bar{\vartheta_{0}}\left(ℑ,\varsigma ,℘\right)+\bar{\vartheta_{1}}\left(ℑ,\varsigma ,℘\right)+\bar{\vartheta_{2}}\left(ℑ,\varsigma ,℘\right)+\bar{\vartheta_{3}}\left(ℑ,\varsigma ,℘\right)+\cdots ,\\ & =\bar{k}e^{-(ℑ+\varsigma )}+ℑ\frac{{℘}^{\beta }}{\beta !}+\varsigma \frac{{℘}^{\beta }}{\beta !}+\frac{{℘}^{\beta +1}}{(\beta +1)!}+2\bar{k}e^{-(ℑ+\varsigma )}\frac{{℘}^{\beta }}{\beta !}+4\bar{k}e^{-(ℑ+\varsigma )}\frac{{℘}^{2\beta }}{(2\beta )!}+8\bar{k}e^{-(ℑ+\varsigma )}\frac{{℘}^{3\beta }}{(3\beta )!}+\cdots . \end{array} \end{array}$$ (42)

**Remark:** If g(ℑ,ς,℘)=0, then above equation becomes as

$$\begin{array}{c} \bar{\vartheta }\left(ℑ,\varsigma ,℘\right)=\bar{k}e^{-(ℑ+\varsigma )}+2\bar{k}e^{-(ℑ+\varsigma )}\frac{{℘}^{\beta }}{\beta !}+4\bar{k}e^{-(ℑ+\varsigma )}\frac{{℘}^{2\beta }}{(2\beta )!}+8\bar{k}e^{-(ℑ+\varsigma )}\frac{{℘}^{3\beta }}{(3\beta )!}+\cdots . \end{array}$$ (43)

In close form, it turns

$$\begin{array}{c} \bar{\vartheta }\left(ℑ,\varsigma ,℘\right)=\bar{k}e^{-(ℑ+\varsigma )}\sum_{n=0}^{\infty }\frac{{\left(2{℘}^{\beta }\right)}^{n}}{(n\beta )!}. \end{array}$$ (44)

**Fig 1:**
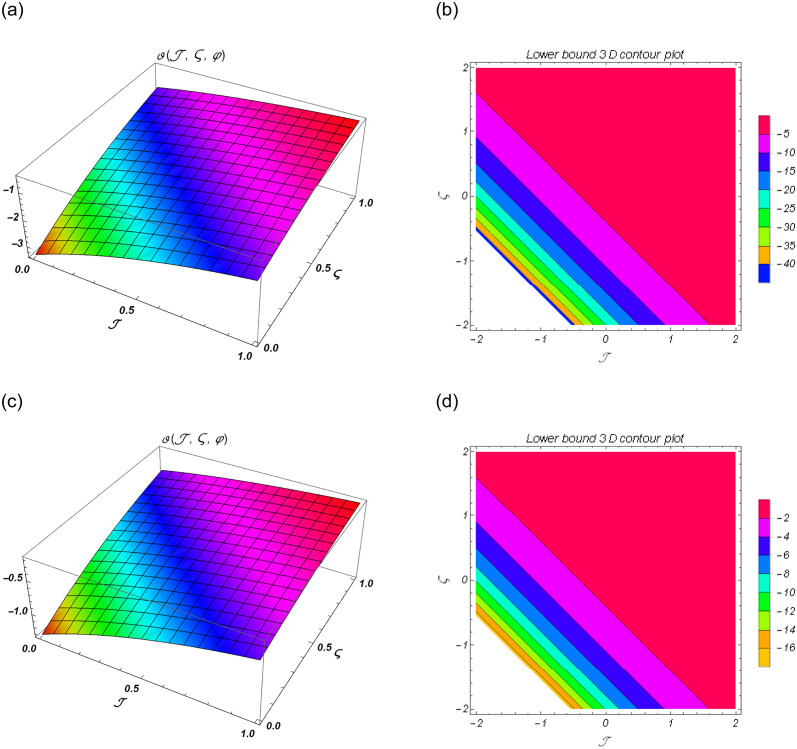
The 3D surface and contour plots for lower bound solutions at *α* = 1 of Example 1. (a) Surface plot of ϑ(ℑ,ς,℘) at *α* = 0.5, (b) Contour plot of ϑ(ℑ,ς,℘) at *α* = 0.5, (c) Surface plot of ϑ(ℑ,ς,℘) at *α* = 1, (d) Contour plot of ϑ(ℑ,ς,℘) at *α* = 1.

**Fig 2:**
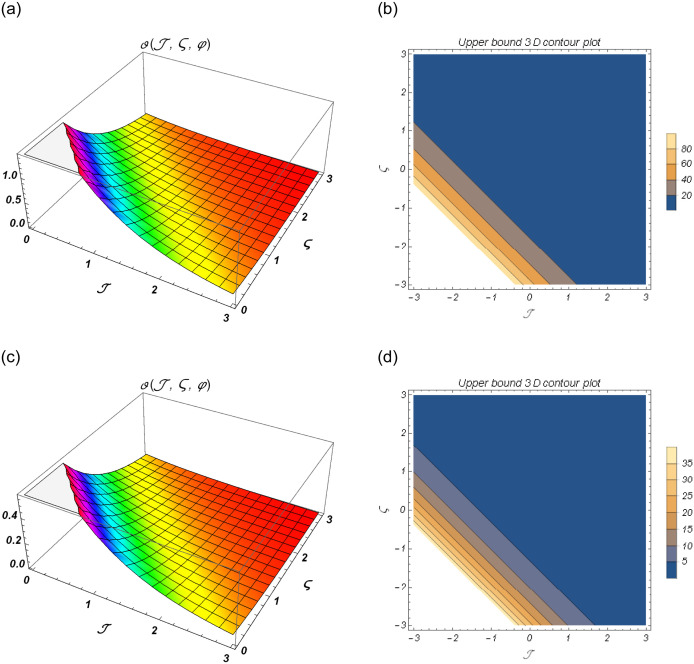
The 3D surface and contour plots for upper bound solutions at *β* = 1 of Example 1. (a) Surface plot of ϑ(ℑ,ς,℘) at *β* = 0.5, (b) Contour plot of ϑ(ℑ,ς,℘) at *β* = 0.5, (c) Surface plot of ϑ(ℑ,ς,℘) at *β* = 1, (d) Contour plot of ϑ(ℑ,ς,℘) at *β* = 1.

**Fig 3:**
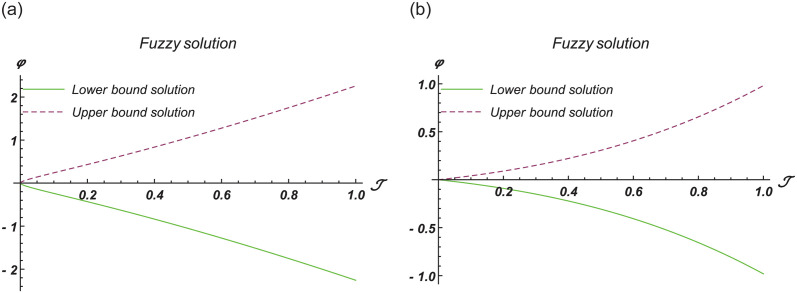
2D fuzzy lower and upper bound solutions at different fractional order of Example 1. (a) 2D fuzzy plot of ϑ(ℑ,ς,℘) at *α* = *β* = 0.5, (b) 2D fuzzy plot of ϑ(ℑ,ς,℘) at *α* = *β* = 1.

Fig 1(a)–1(d) show the lower bound fuzzy results at different fractional order of *α*. Fig 1(a) and 1(c) shows the fuzzy surface solutions with space coordinates *r* = 0.5, 0 ≤ ℑ ≤ 1, 0≤ς≤1 where the fractional orders are *α* = 0.5 and *α* = 1. On the other hand, Fig 1(b) and 1(d) shows the fuzzy contour solutions with space coordinates **℘** = 0.1, *r* = 0.5, −2 ≤ ℑ ≤ 2, -2≤ς≤2 where the fractional orders are *α* = 0.5 and *α* = 1. Fig 2(a) and 2(d) show the upper bound fuzzy results at different fractional order of *α*. Fig 2(a) and 2(c) shows the fuzzy surface solutions with space coordinates *r* = 0.5, 0 ≤ ℑ ≤ 3, 0≤ς≤3 where the fractional orders are *α* = 0.5 and *α* = 1. On the other hand, Fig 2(b) and 2(d) shows the fuzzy surface solutions with space coordinates **℘** = 0.1, *r* = 0.5, −3 ≤ ℑ ≤ 3, -3≤ς≤3 where the fractional orders are *α* = 0.5 and *α* = 1. Fig 3(a) and 3(b) demonstrate the 2D representation at *α* = 0.5 and *α* = 1 respectively.

### 6.2 Example 2

Again, we assume 2D homogeneous heat flow problem in fractional order as

$$\begin{array}{c} D_{℘}^{\alpha ,\;\beta }\tilde{\vartheta }\left(ℑ,\varsigma ,℘\right)={\tilde{\vartheta }}_{ℑℑ}\left(ℑ,\varsigma ,℘\right)+{\tilde{\vartheta }}_{\varsigma \varsigma }\left(ℑ,\varsigma ,℘\right)+ℑ+\varsigma +{℘}^{2}, \end{array}$$ (45)

with the initial condition

$$\begin{array}{c} \tilde{\vartheta }\left(ℑ,\varsigma ,0\right)=\tilde{k}\;\text{sin}\left[\pi \left(ℑ+\varsigma \right)\right]. \end{array}$$ (46)

where $\tilde{k}=\left[\underline{k},\bar{k}\right]=\left[r-1,1-r\right]$.

#### 6.2.1 For lower bound solution

Since, we have

$$\begin{array}{c} \frac{\partial^{\alpha }\underline{\vartheta }}{\partial {℘}^{\alpha }}=\frac{\partial^{2}\underline{\vartheta }}{\partial {ℑ}^{2}}+\frac{\partial^{2}\underline{\vartheta }}{\partial \varsigma^{2}}+ℑ+\varsigma +{℘}^{2}, \end{array}$$ (47)

subjected to the condition

$$\begin{array}{c} \underline{\vartheta }\left(ℑ,\varsigma ,0\right)=\underline{k}\;\text{sin}\left[\pi \left(ℑ+\varsigma \right)\right]. \end{array}$$ (48)

where $\underline{k}=r-1$.

Apply ST on Eq (47), we get

$$\begin{array}{c} S[\frac{\partial^{\alpha }\underline{\vartheta }}{\partial {℘}^{\alpha }}]=S[\frac{\partial^{2}\underline{\vartheta }}{\partial {ℑ}^{2}}+\frac{\partial^{2}\underline{\vartheta }}{\partial \varsigma^{2}}+ℑ+\varsigma +{℘}^{2}]. \end{array}$$

Using the Sawi transform in fractional derivative, we obtain

$$\begin{array}{c} S\left[\underline{\vartheta }\left(ℑ,\varsigma ,℘\right)\right]=\frac{1}{\theta }\underline{\vartheta }\left(ℑ,\varsigma ,0\right)+\theta^{\alpha }S[\frac{\partial^{2}\underline{\vartheta }}{\partial {ℑ}^{2}}+\frac{\partial^{2}\underline{\vartheta }}{\partial \varsigma^{2}}+ℑ+\varsigma +{℘}^{2}]. \end{array}$$

In other way, we can also write it as

$$\begin{array}{c} S\left[\underline{\vartheta }\left(ℑ,\varsigma ,℘\right)\right]=\frac{1}{\theta }\underline{\vartheta }\left(ℑ,\varsigma ,0\right)+\theta^{\alpha }S\left[ℑ+\varsigma +{℘}^{2}\right]+\theta^{\alpha }S[\frac{\partial^{2}\underline{\vartheta }}{\partial {ℑ}^{2}}+\frac{\partial^{2}\underline{\vartheta }}{\partial \varsigma^{2}}]. \end{array}$$

Using inverse ST, we get

$$\begin{array}{c} \vartheta \left(ℑ,\varsigma ,℘\right)=\underline{\vartheta }\left(ℑ,\varsigma ,0\right)+ℑ\frac{{℘}^{\alpha }}{\alpha !}+\varsigma \frac{{℘}^{\alpha }}{\alpha !}+2\frac{{℘}^{\alpha +2}}{(\alpha +2)!}+S^{-1}[\theta^{\alpha }S[\frac{\partial^{2}\underline{\vartheta }}{\partial {ℑ}^{2}}+\frac{\partial^{2}\underline{\vartheta }}{\partial \varsigma^{2}}]. \end{array}$$ (49)

Implement the idea of HPS on Eq (49), we obtain the He’s iterations such as

$$\begin{array}{c} \sum_{i=0}^{\infty }p^{i}\underline{\vartheta }\left(ℑ,\varsigma ,℘\right)=\underline{\vartheta }\left(ℑ,\varsigma ,0\right)+ℑ\frac{{℘}^{\alpha }}{\alpha !}+\varsigma \frac{{℘}^{\alpha }}{\alpha !}+2\frac{{℘}^{\alpha +2}}{(\alpha +2)!}+S^{-1}[\theta^{\alpha }S[\sum_{i=0}^{\infty }p^{i}\frac{\partial^{2}\underline{\vartheta_{i}}}{\partial {ℑ}^{2}}+\sum_{i=0}^{\infty }p^{i}\frac{\partial^{2}\underline{\vartheta_{i}}}{\partial \varsigma^{2}}]. \end{array}$$

By comparing the related factors of *p*, we obtain

$$\begin{array}{cc} p^{0} & :\underline{\vartheta_{0}}\left(ℑ,\varsigma ,℘\right)=\underline{\vartheta }\left(ℑ,\varsigma ,0\right)=\underline{k}\;\text{sin}\left[\pi \left(ℑ+\varsigma \right)\right]+ℑ\frac{{℘}^{\alpha }}{\alpha !}+\varsigma \frac{{℘}^{\alpha }}{\alpha !}+2\frac{{℘}^{\alpha +2}}{(\alpha +2)!},\\ p^{1} & :\underline{\vartheta_{1}}\left(ℑ,\varsigma ,℘\right)=S^{-1}[\theta^{\alpha }S\{\frac{\partial^{2}\underline{\vartheta_{0}}}{\partial {ℑ}^{2}}+\frac{\partial^{2}\underline{\vartheta_{0}}}{\partial \varsigma^{2}}\}]=-2\underline{k}\pi^{2}\text{sin}\left[\pi \left(ℑ+\varsigma \right)\right]\frac{{℘}^{\alpha }}{\alpha !},\\ p^{2} & :\underline{\vartheta_{2}}\left(ℑ,\varsigma ,℘\right)=S^{-1}[\theta^{\alpha }S\{\frac{\partial^{2}\underline{\vartheta_{1}}}{\partial {ℑ}^{2}}+\frac{\partial^{2}\underline{\vartheta_{1}}}{\partial \varsigma^{2}}\}]=4\underline{k}\pi^{2}\text{sin}\left[\pi \left(ℑ+\varsigma \right)\right]\frac{{℘}^{2\alpha }}{(2\alpha )!},\\ p^{3} & :\underline{\vartheta_{3}}\left(ℑ,\varsigma ,℘\right)=S^{-1}[\theta^{\alpha }S\{\frac{\partial^{2}\underline{\vartheta_{2}}}{\partial {ℑ}^{2}}+\frac{\partial^{2}\underline{\vartheta_{2}}}{\partial \varsigma^{2}}\}]=-8\underline{k}\pi^{2}\text{sin}\left[\pi \left(ℑ+\varsigma \right)\right]\frac{{℘}^{3\alpha }}{(3\alpha )!},\\ & \vdots . \end{array}$$

In other words

$$\begin{array}{c} \begin{array}{cc} \underline{\vartheta }\left(ℑ,\varsigma ,℘\right) & =\underline{\vartheta_{0}}\left(ℑ,\varsigma ,℘\right)+\underline{\vartheta_{1}}\left(ℑ,\varsigma ,℘\right)+\underline{\vartheta_{2}}\left(ℑ,\varsigma ,℘\right)+\underline{\vartheta_{3}}\left(ℑ,\varsigma ,℘\right)+\cdots ,\\ & =\underline{k}\;\text{sin}\left[\pi \left(ℑ+\varsigma \right)\right]+ℑ\frac{{℘}^{\alpha }}{\alpha !}+\varsigma \frac{{℘}^{\alpha }}{\alpha !}+2\frac{{℘}^{\alpha +2}}{(\alpha +2)!}-2\underline{k}\pi^{2}\text{sin}\left[\pi \left(ℑ+\varsigma \right)\right]\frac{{℘}^{\alpha }}{\alpha !}\\ & +4\underline{k}\pi^{2}\text{sin}\left[\pi \left(ℑ+\varsigma \right)\right]\frac{{℘}^{2\alpha }}{(2\alpha )!}-8\underline{k}\pi^{2}\text{sin}\left[\pi \left(ℑ+\varsigma \right)\right]\frac{{℘}^{3\alpha }}{(3\alpha )!}+\cdots . \end{array} \end{array}$$ (50)

**Remark:** If g(ℑ,ς,℘)=0, then above equation becomes as

$$\begin{array}{c} \begin{array}{cc} \underline{\vartheta }\left(ℑ,\varsigma ,℘\right) & =\underline{k}\text{sin}\left[\pi \left(ℑ+\varsigma \right)\right]-2\underline{k}\pi^{2}\text{sin}\left[\pi \left(ℑ+\varsigma \right)\right]\frac{{℘}^{\alpha }}{\alpha !}+4\underline{k}\pi^{4}\text{sin}\left[\pi \left(ℑ+\varsigma \right)\right]\frac{{℘}^{2\alpha }}{(2\alpha )!}\\ & -8\underline{k}\pi^{6}\text{sin}\left[\pi \left(ℑ+\varsigma \right)\right]\frac{{℘}^{3\alpha }}{(3\alpha )!}+\cdots . \end{array} \end{array}$$ (51)

In close form, it turns

$$\begin{array}{c} \underline{\vartheta }\left(ℑ,\varsigma ,℘\right)=\underline{k}\text{sin}\left[\pi \left(ℑ+\varsigma \right)\right]\sum_{n=0}^{\infty }\frac{{\left(-1\right)}^{n}{\left(2\pi^{2}{℘}^{\alpha }\right)}^{n}}{(n\alpha )!}. \end{array}$$ (52)

#### 6.2.2 For upper bound solution

Since, we have

$$\begin{array}{c} \frac{\partial^{\beta }\bar{\vartheta }}{\partial {℘}^{\beta }}=\frac{\partial^{2}\bar{\vartheta }}{\partial {ℑ}^{2}}+\frac{\partial^{2}\bar{\vartheta }}{\partial \varsigma^{2}}+ℑ+\varsigma +{℘}^{2}, \end{array}$$ (53)

with the initial condition

$$\begin{array}{c} \bar{\vartheta }\left(ℑ,\varsigma ,0\right)=\bar{k}\text{sin}\left[\pi \left(ℑ+\varsigma \right)\right]. \end{array}$$ (54)

where $\bar{k}=1-r$.

Apply ST on Eq (53), we get

$$\begin{array}{c} S[\frac{\partial^{\beta }\bar{\vartheta }}{\partial {℘}^{\beta }}]=S[\frac{\partial^{2}\bar{\vartheta }}{\partial {ℑ}^{2}}+\frac{\partial^{2}\bar{\vartheta }}{\partial \varsigma^{2}}+ℑ+\varsigma +{℘}^{2}]. \end{array}$$ (55)

Using the Sawi transform in fractional derivative, we obtain

$$\begin{array}{c} S\left[\bar{\vartheta }\left(ℑ,\varsigma ,℘\right)\right]=\frac{1}{\theta }\bar{\vartheta }\left(ℑ,\varsigma ,0\right)+\theta^{\beta }S[\frac{\partial^{2}\bar{\vartheta }}{\partial {ℑ}^{2}}+\frac{\partial^{2}\bar{\vartheta }}{\partial \varsigma^{2}}+ℑ+\varsigma +{℘}^{2}]. \end{array}$$

In other way, we can also write it as

$$\begin{array}{c} S\left[\bar{\vartheta }\left(ℑ,\varsigma ,℘\right)\right]=\frac{1}{\theta }\bar{\vartheta }\left(ℑ,\varsigma ,0\right)+\theta^{\beta }S\left[ℑ+\varsigma +{℘}^{2}\right]+\theta^{\beta }S[\frac{\partial^{2}\bar{\vartheta }}{\partial {ℑ}^{2}}+\frac{\partial^{2}\bar{\vartheta }}{\partial \varsigma^{2}}]. \end{array}$$

Using inverse ST, we get

$$\begin{array}{c} \vartheta \left(ℑ,\varsigma ,℘\right)=\bar{\vartheta }\left(ℑ,\varsigma ,0\right)+ℑ\frac{{℘}^{\beta }}{\beta !}+\varsigma \frac{{℘}^{\beta }}{\beta !}+2\frac{{℘}^{\beta +2}}{(\beta +2)!}+S^{-1}[\theta^{\beta }S[\frac{\partial^{2}\bar{\vartheta }}{\partial {ℑ}^{2}}+\frac{\partial^{2}\bar{\vartheta }}{\partial \varsigma^{2}}]. \end{array}$$ (56)

Implement the idea of HPS on Eq (56), we obtain the He’s iterations such as

$$\begin{array}{c} \sum_{i=0}^{\infty }p^{i}\bar{\vartheta }\left(ℑ,\varsigma ,℘\right)=\bar{\vartheta }\left(ℑ,\varsigma ,0\right)+ℑ\frac{{℘}^{\beta }}{\beta !}+\varsigma \frac{{℘}^{\beta }}{\beta !}+2\frac{{℘}^{\beta +2}}{(\beta +2)!}+S^{-1}[\theta^{\beta }S[\sum_{i=0}^{\infty }p^{i}\frac{\partial^{2}\underline{\vartheta_{i}}}{\partial {ℑ}^{2}}+\sum_{i=0}^{\infty }p^{i}\frac{\partial^{2}\underline{\vartheta_{i}}}{\partial \varsigma^{2}}]. \end{array}$$

By examining the related factors of *p*, we arrive at

$$\begin{array}{cc} p^{0} & :\underline{\vartheta_{0}}\left(ℑ,\varsigma ,℘\right)=\bar{\vartheta }\left(ℑ,\varsigma ,0\right)=\bar{k}\text{sin}\left[\pi \left(ℑ+\varsigma \right)\right]+ℑ\frac{{℘}^{\beta }}{\beta !}+\varsigma \frac{{℘}^{\beta }}{\beta !}+2\frac{{℘}^{\beta +2}}{(\beta +2)!},\\ p^{1} & :\underline{\vartheta_{1}}\left(ℑ,\varsigma ,℘\right)=S^{-1}[\theta^{\beta }S\{\frac{\partial^{2}\underline{\vartheta_{0}}}{\partial {ℑ}^{2}}+\frac{\partial^{2}\underline{\vartheta_{0}}}{\partial \varsigma^{2}}\}]=-2\bar{k}\pi^{2}\text{sin}\left[\pi \left(ℑ+\varsigma \right)\right]\frac{{℘}^{\beta }}{\beta !},\\ p^{2} & :\underline{\vartheta_{2}}\left(ℑ,\varsigma ,℘\right)=S^{-1}[\theta^{\beta }S\{\frac{\partial^{2}\underline{\vartheta_{1}}}{\partial {ℑ}^{2}}+\frac{\partial^{2}\underline{\vartheta_{1}}}{\partial \varsigma^{2}}\}]=4\bar{k}\pi^{2}\text{sin}\left[\pi \left(ℑ+\varsigma \right)\right]\frac{{℘}^{2\beta }}{(2\beta )!},\\ p^{3} & :\underline{\vartheta_{3}}\left(ℑ,\varsigma ,℘\right)=S^{-1}[\theta^{\beta }S\{\frac{\partial^{2}\underline{\vartheta_{2}}}{\partial {ℑ}^{2}}+\frac{\partial^{2}\underline{\vartheta_{2}}}{\partial \varsigma^{2}}\}]=-8\bar{k}\pi^{2}\text{sin}\left[\pi \left(ℑ+\varsigma \right)\right]\frac{{℘}^{3\beta }}{(3\beta )!},\\ & \vdots . \end{array}$$

In other words

$$\begin{array}{c} \begin{array}{cc} \bar{\vartheta }\left(ℑ,\varsigma ,℘\right) & =\underline{\vartheta_{0}}\left(ℑ,\varsigma ,℘\right)+\underline{\vartheta_{1}}\left(ℑ,\varsigma ,℘\right)+\underline{\vartheta_{2}}\left(ℑ,\varsigma ,℘\right)+\underline{\vartheta_{3}}\left(ℑ,\varsigma ,℘\right)+\cdots ,\\ & =\bar{k}\text{sin}\left[\pi \left(ℑ+\varsigma \right)\right]+ℑ\frac{{℘}^{\beta }}{\beta !}+\varsigma \frac{{℘}^{\beta }}{\beta !}+2\frac{{℘}^{\beta +2}}{(\beta +2)!}-2\bar{k}\pi^{2}\text{sin}\left[\pi \left(ℑ+\varsigma \right)\right]\frac{{℘}^{\beta }}{\beta !}\\ & +4\bar{k}\pi^{4}\text{sin}\left[\pi \left(ℑ+\varsigma \right)\right]\frac{{℘}^{2\beta }}{(2\beta )!}-8\bar{k}\pi^{6}\text{sin}\left[\pi \left(ℑ+\varsigma \right)\right]\frac{{℘}^{3\beta }}{(3\beta )!}+\cdots . \end{array} \end{array}$$ (57)

**Remark:** If g(ℑ,ς,℘)=0, then above equation becomes as

$$\begin{array}{c} \begin{array}{cc} \bar{\vartheta }\left(ℑ,\varsigma ,℘\right) & =\bar{k}\text{sin}\left[\pi \left(ℑ+\varsigma \right)\right]-2\bar{k}\pi^{2}\text{sin}\left[\pi \left(ℑ+\varsigma \right)\right]\frac{{℘}^{\beta }}{\beta !}+4\bar{k}\pi^{2}\text{sin}\left[\pi \left(ℑ+\varsigma \right)\right]\frac{{℘}^{2\beta }}{(2\beta )!}\\ & -8\bar{k}\pi^{2}\text{sin}\left[\pi \left(ℑ+\varsigma \right)\right]\frac{{℘}^{3\beta }}{(3\beta )!}+\cdots . \end{array} \end{array}$$ (58)

In close form, it turns

$$\begin{array}{c} \bar{\vartheta }\left(ℑ,\varsigma ,℘\right)=\bar{k}\text{sin}\left[\pi \left(ℑ+\varsigma \right)\right]\sum_{n=0}^{\infty }\frac{{\left(-1\right)}^{n}{\left(2\pi^{2}{℘}^{\beta }\right)}^{n}}{(n\beta )!}. \end{array}$$ (59)

**Fig 4:**
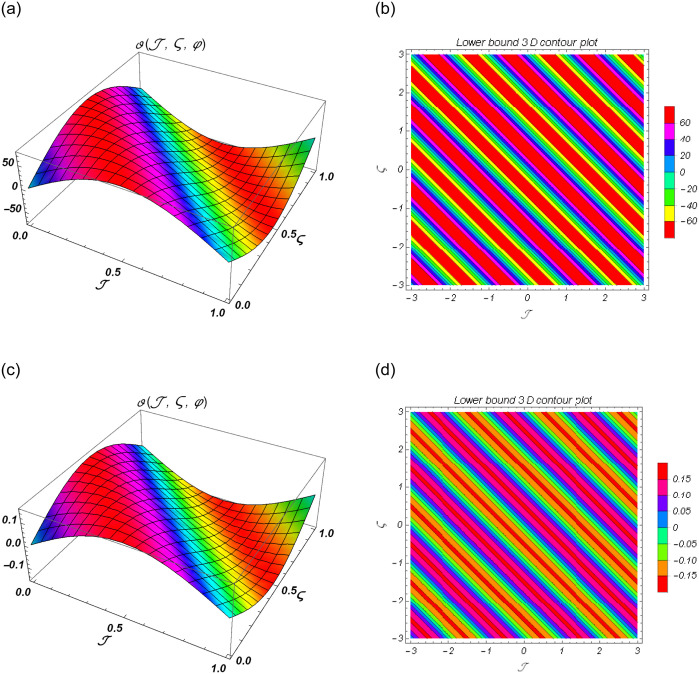
The 3D surface and contour plots for lower bound solutions at *α* = 1 of Example 2. (a) Surface plot of ϑ(ℑ,ς,℘) at *α* = 0.5, (b) Contour plot of ϑ(ℑ,ς,℘) at *α* = 0.5, (c) Surface plot of ϑ(ℑ,ς,℘) at *α* = 1, (d) Contour plot of ϑ(ℑ,ς,℘) at *α* = 1.

**Fig 5:**
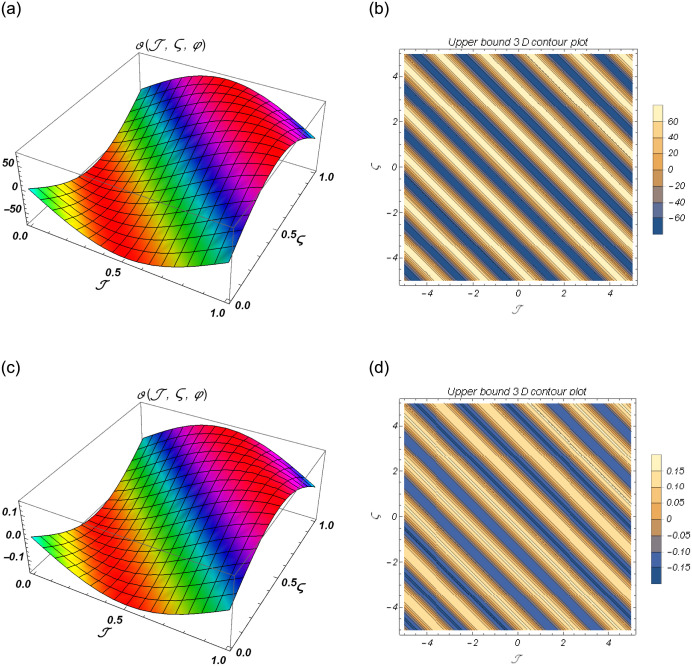
The 3D surface and contour plots for upper bound solutions at *β* = 1 of Example 2. (a) Surface plot of ϑ(ℑ,ς,℘) at *β* = 0.5, (b) Contour plot of ϑ(ℑ,ς,℘) at *β* = 0.5, (c) Surface plot of ϑ(ℑ,ς,℘) at *β* = 1, (d) Contour plot of ϑ(ℑ,ς,℘) at *β* = 1.

**Fig 6:**
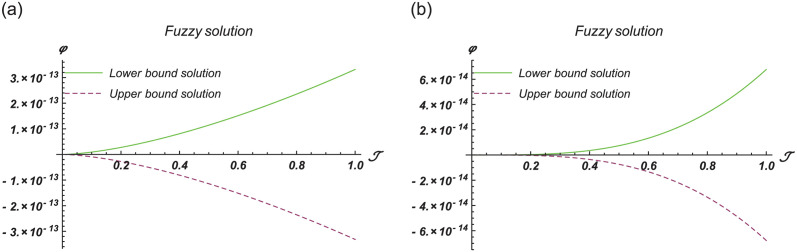
2D fuzzy lower and upper bound solutions at different fractional order of Example 2. (a) 2D fuzzy plot of ϑ(ℑ,ς,℘) at *α* = *β* = 0.5, (b) 2D fuzzy plot of ϑ(ℑ,ς,℘) at *α* = *β* = 1.

Fig 4(a)–4(d) show the lower bound fuzzy results at different fractional order of *α*. Fig 4(a) and 4(c) shows the fuzzy surface solutions with space coordinates *r* = 0.5, 0 ≤ ℑ ≤ 1, 0≤ς≤1 where the fractional orders are *α* = 0.5 and *α* = 1. On the other hand, Fig 4(b) and 4(d) shows the fuzzy contour solutions with space coordinates **℘** = 0.1, *r* = 0.5, −3 ≤ ℑ ≤ 3, -3≤ς≤3 where the fractional orders are *α* = 0.5 and *α* = 1. Fig 5(a) and 5(d) show the upper bound fuzzy results at different fractional order of *α*. Fig 5(a) and 5(c) shows the fuzzy surface solutions with space coordinates *r* = 0.5, 0 ≤ ℑ ≤ 1, 0≤ς≤1 where the fractional orders are *α* = 0.5 and *α* = 1. On the other hand, Fig 5(b) and 5(d) shows the fuzzy surface solutions with space coordinates **℘** = 0.1, *r* = 0.5, −5 ≤ ℑ ≤ 5, -5≤ς≤5 where the fractional orders are *α* = 0.5 and *α* = 1. Fig 6(a) and 6(b) demonstrate the 2D representation at *α* = 0.5 and *α* = 1 respectively.

## 7 Conclusion

In this research, we construct the Sawi homotopy perturbation transform scheme (SHPTS) for the approximate solution of two-dimensional fuzzy fractional heat equation. The obtained results in terms of series show the validity and accuracy of this proposed scheme. The contour and surface representations are offered for the lower and upper-bound solutions. By demonstrating the surface and contour plots for two-dimensional fuzzy fractional heat equation, the correctness and capabilities of the proposed algorithm is showed. We provide the surface and contour representations for both the upper- and lower-bound solutions. It has been proved that the suggested framework will allow it to work with fuzzy fractional partial differential equations in various dimensions. In further study, this strategy may be utilized to provide analytical and approximation results for unstable fractional differential equations under instability with non-classical and integral boundary scenarios in the context of Caputo-Fabrizio.

## Supporting information

S1 File (DOCX)
